# Metastatic breast cancer with leptomeningeal carcinomatosis treated with trastuzumab deruxtecan – a case report

**DOI:** 10.18632/oncoscience.631

**Published:** 2025-10-09

**Authors:** Cristiana Honrado Martins, José Miguel Rocha, Catarina Portela, Ricardo Fernandes, Cláudia Caeiro

**Affiliations:** ^1^Medical Oncology Resident, Medical Oncology Department, Hospital de Braga, ULS de Braga, Braga 4710-243, Portugal; ^2^Medical Oncology Specialist, Medical Oncology Department, Hospital de Braga, ULS de Braga, Braga 4710-243, Portugal

**Keywords:** breast cancer, leptomeningeal carcinomatosis, HER2 positive, antibody-drug conjugate, trastuzumab-deruxtecan

## Abstract

Breast cancer (BC) is the most common malignancy in women. Both histology and immunochemistry are core to treatment choice and the knowledge of these fields is expanding. Metastatic breast cancer (MBC) has significantly lower overall survival (OS) than early stages, and leptomeningeal carcinomatosis (LMC) and brain metastasis (BM) have particularly worse prognosis.

We describe a case of LMC BC treated with trastuzumab-deruxtecan (T-DXd). Thirty-seven years old woman, diagnosed with a HER2-positive invasive lobular carcinoma (cT2N0M0). After initial treatment with neoadjuvant chemotherapy plus anti-HER2 double blockade, surgery and postoperative radiotherapy, the patient relapsed with *de novo* LMC and BM, two years after initial diagnosis. Systemic therapy with first line off-label T-DXd was initiated, resulting in disease response, neurological recovery and improved quality of life (QoL).

Disease complexity and expansion of new therapeutic options in BC has made multidisciplinary team discussion mandatory. In the HER2-positive MBC setting, antibody-drug conjugates (ADC), such as trastuzumab-emtansine and T-DXd, have shown important disease outcomes and QoL improvement. Equally important, these treatments are well tolerated and have manageable adverse events, making them safe and effective drugs. This solid evidence is seemingly leading into a new and groundbreaking BC treatment era.

## INTRODUCTION

Breast cancer (BC) is the most frequent malignancy affecting women. The incidence of new BC cases is rising, accounting for 11.7% of new tumor diagnosis in 2020 [1]. However, in developed countries, BC mortality has been declining, due to better screening, diagnosis and treatment [2].

BC is a highly heterogeneous disease. Over time, new discoveries relating to BC histology and immunochemistry (IHQ) allowed a better perception of molecular and biological behavior. Knowledge about hormonal receptors (HR) (estrogen receptor (ER) and progesterone receptor (PR)), activation of human epidermal growth factor receptor 2 (HER2), gene mutations (e.g., BRCA 1/2) and biomarkers of immune environment (e.g., tumor-infiltrating lymphocytes (TILs) and programmed death-ligand 1 (PD-L1)) are key to best treatment choices and long-term prognosis [3].

Metastatic breast cancer (MBC) is an incurable disease, with significantly lower overall survival (OS) rates when compared to early breast cancer (EBC) [4]. Leptomeningeal carcinomatosis (LMC), as well as brain metastasis (BM), are relatively common in triple-negative and HER2-positive cases [5, 6], representing a worse prognosis factor. Nevertheless, new therapeutic approaches have been developed as MBC standard-of-care, leading to OS improvements [4].

The rising of new targeted drugs in BC, especially the advent of antibody-drug conjugates (ADC), is leading to a new era. Approval of trastuzumab emtansine (T-DM1) and trastuzumab deruxtecan (T-DXd) for HER2-positive MBC treatment, based on significantly increasing OS data [7, 8], are examples of a major therapeutic breakthrough.

In this report, we describe a case of leptomeningeal carcinomatosis from breast cancer treated with T-DXd.

## CASE PRESENTATION

We describe the case of a 37-year-old woman, with no relevant past medical history or family background. The patient was referred to our center due to a right nipple retraction with a stiff right retroareolar mass (3.5 cm of largest diameter), noted during breast auto-examination three months prior. Axillary nodes were not palpable.

Ultrasound guided biopsy confirmed histological diagnosis of invasive lobular carcinoma, G2, HR negative, HER2-positive, ki67 of 30%. Bilateral breast magnetic resonance imaging (b-MRI) showed no other suspect breast or axillary lesions. Staging for distant metastatic disease was negative (cT2N0M0). Tumor markers (CEA and C.A. 15.3) were negative. No germline mutations were identified.

Neoadjuvant chemotherapy (CTx) was decided (dose-dense doxorubicin and cyclophosphamide followed by weekly paclitaxel and dual anti-HER2 blockade with trastuzumab/pertuzumab), followed by right lumpectomy and selective sentinel node biopsy. Pathological complete response (CR) was achieved (ypT0 N0(sn)). Postoperative radiotherapy (RT) to the breast was performed afterwards. After that, a surveillance protocol was initiated.

**Figure 1:**
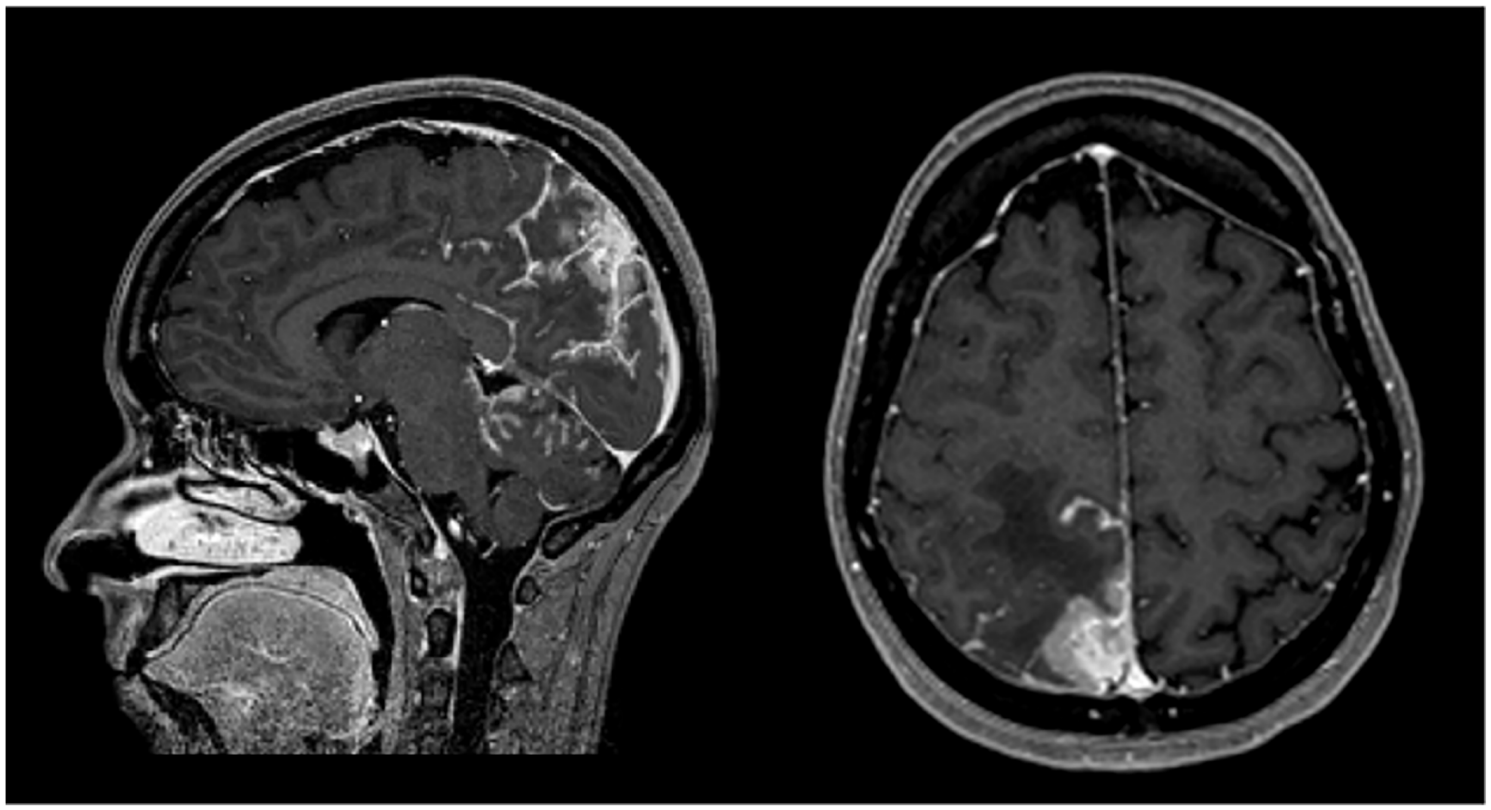
CE MRI at BM and LMC initial moment of diagnosis. Heterogeneous space-occupying lesion (right posterior parietal-parasagittal cortico-subcortical area), with vasogenic edema and sulcal effacement, compatible with a BM lesion, along with pachymeningeal carcinomatosis (right next to the first described BM).

**Figure 2:**
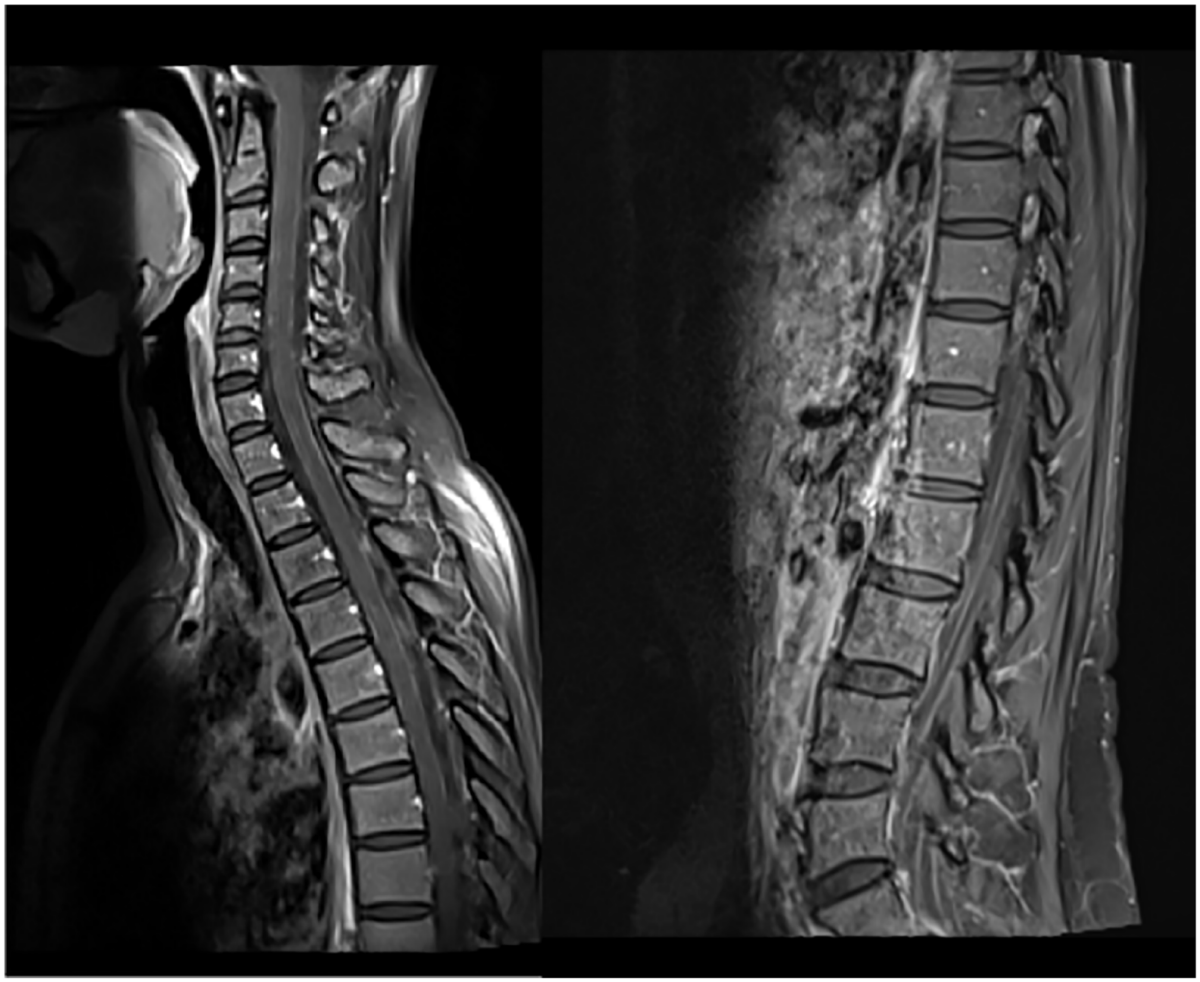
Neuraxis MRI at BM and LMC initial moment of diagnosis. Disseminated LMC (extended to cerebellar fissures, midbrain, brachiocephalic bridge and cranial nerves emergence).

Sixteen months later (1.3 years), the patient was admitted to the emergency department with severe right headache (only mild response to pain medication and presence of other red flags: vomiting, night awakening, neurological deficits - visual deficits, facial paresthesias, dizziness; vertigo and decreased left upper limb muscular strength). Cranioencephalic computed tomography (CE-CT) showed a heterogeneous space-occupying lesion, in the right posterior parietal-parasagittal cortico-subcortical area, with vasogenic edema and sulcal effacement, compatible with a new BM lesion. Cranioencephalic magnetic resonance imaging (CE-MRI) confirmed the lesion genesis, showing new pachymeningeal carcinomatosis (right next to the lesion) and disseminated leptomeningeal carcinomatosis (LMC) (extended to cerebellar fissures, midbrain and brachiocephalic bridge, involving the cranial nerves emergence as well) (Figure 1). An important mass effect was present, compressing and occluding the right vertebral artery V4 terminal segment. Neuraxis MRI confirmed disseminated LMC in all neuraxis (extension to the conus medullaris limit) (Figure 2). Lumbar puncture showed a turbid cerebrospinal fluid (CSF), with cytology demonstrating small, scattered lymphocytes, large epithelial cells exhibiting irregular, hyperchromatic nuclei, and the presence of the ‘cell-in-cell’ phenomenon. The fluid was histologically confirmed as central nervous system (CNS) metastasis of HER2 positive BC. Re-staging showed neither local nor distant disease.

Palliative holocranial RT was performed, leading to symptomatic relief. After multidisciplinary and interhospital expert discussion, the patient was proposed to be included in the DEBBRAH trial, which was not possible due to logistical and deadlines events. At this point, a first line systemic therapy with trastuzumab-deruxtecan (T-DXd) in an *off-label* setting was proposed, based upon previous data showing intracranial activity. The drug was started one month after RT ended.

**Figure 3:**
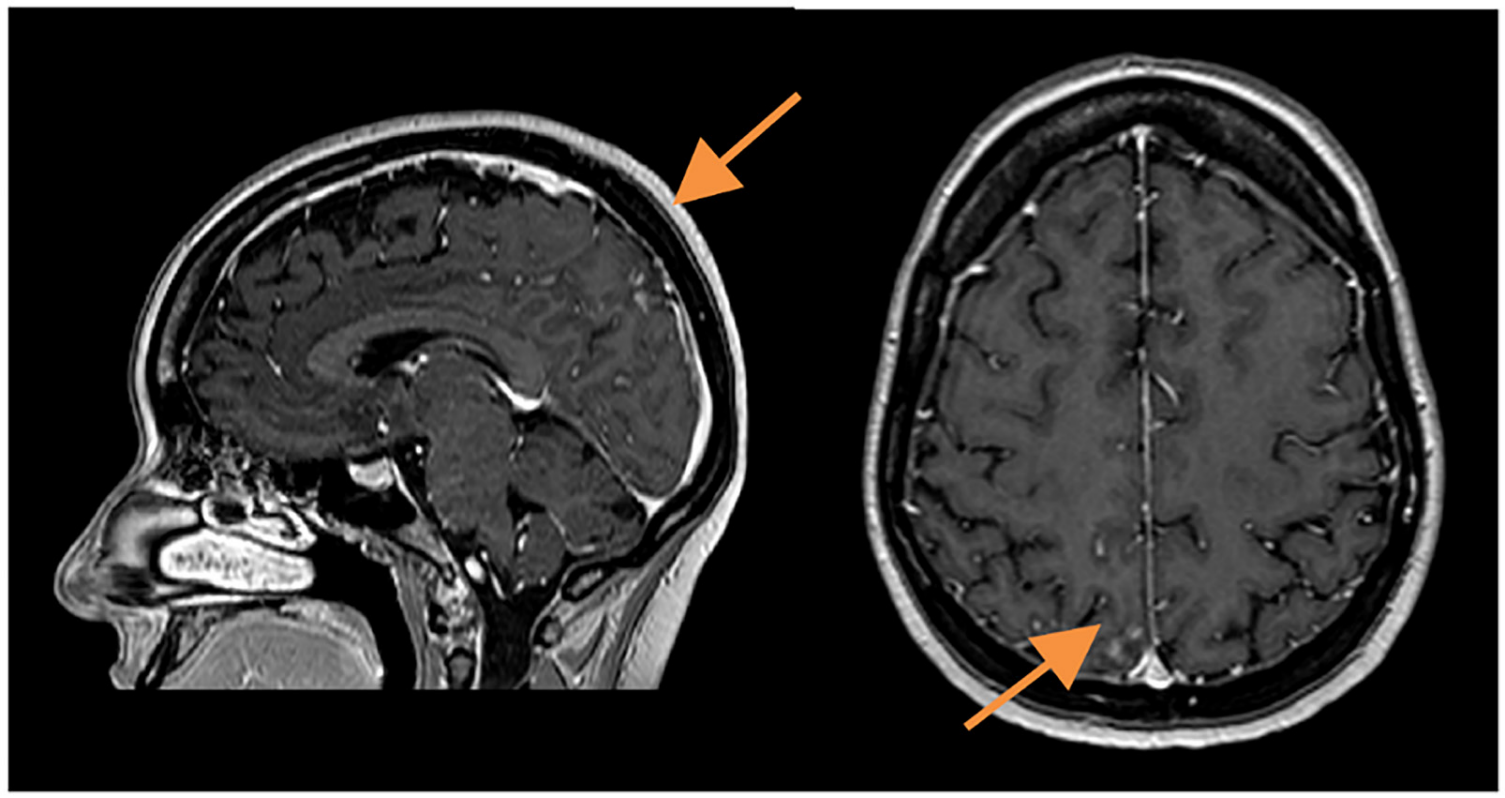
CE MRI response evaluation after 3 cycles of T-DXd. Right parietal lesion was residual and pachymeningeal infiltration was not found (arrows).

**Figure 4:**
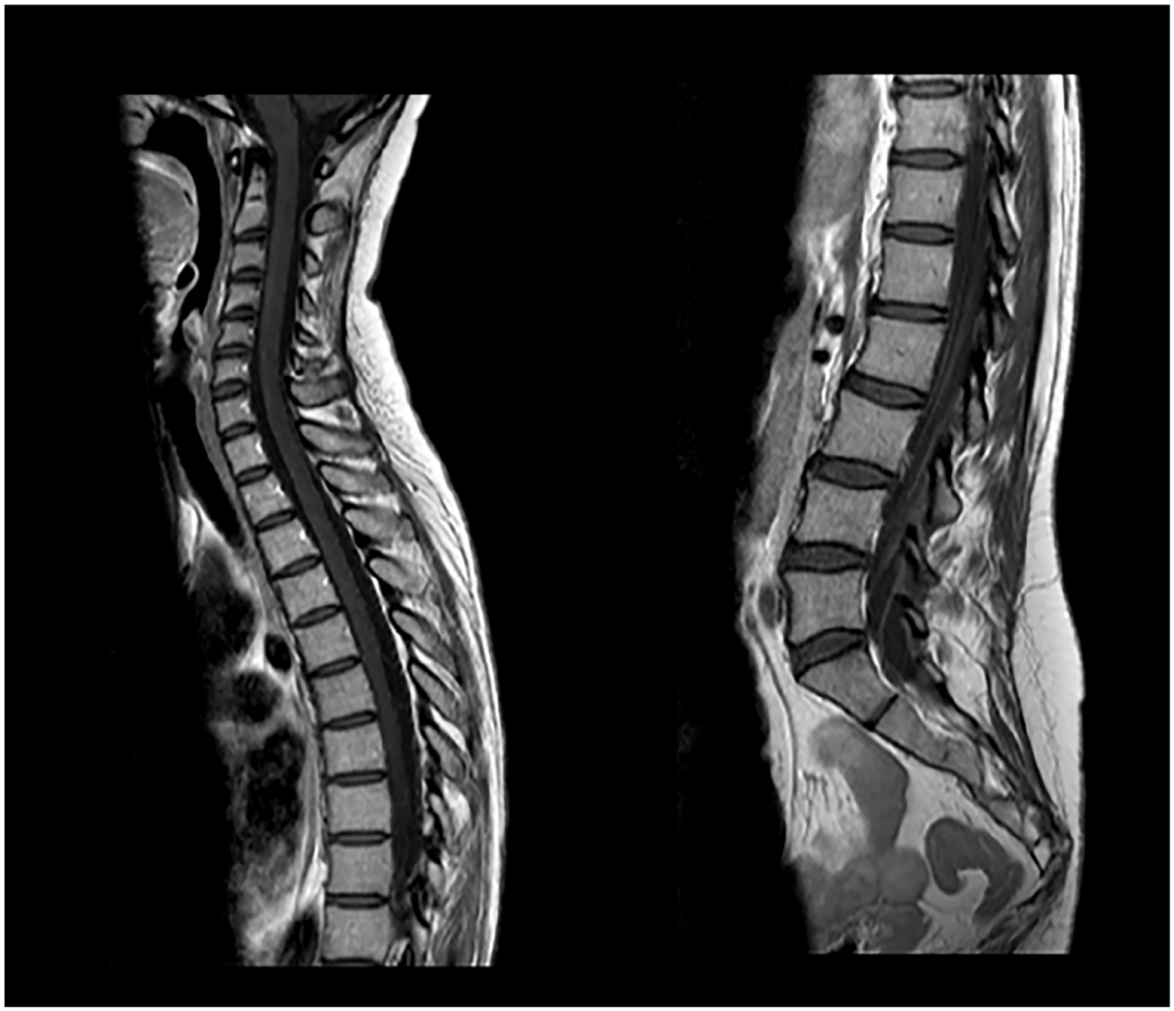
CE and neuraxis MRI response evaluation after 3 cycles of T-DXd. LMC was noticeably reduced (in extension and intensity).

A slow but steady neurological improvement was achieved, with full autonomy recovery after only three cycles of T-DXd. Response evaluation with CE and neuraxis MRI showed excellent disease response: right parietal lesion was residual and pachymeningeal infiltration was not found; LMC was noticeably reduced (in extension and intensity) (Figures 3 and 4). Toraco-abdominal and pelvic CT (TAP-CT) was systematically negative for new distant secondary disease.

The drug was well tolerated, with no grade 4 adverse effects (AE) being observed, according to ‘common terminology criteria for adverse events’ (CTCAE version 5). Grade 1 anemia, emesis, peripheral edemas, palmoplantar hypoesthesias and grade 2 alopecia and urticaria-like rash were easily manageable. Grade 2 thrombocytopenia with epistaxis was also observed, self-limiting and with no other complications. Grade 3 gamma-glutamyl transferase (GGT) dysfunction led to treatment delay until recovery. Serial echocardiograms showed no cardiac toxicity, maintaining a normal left ventricular ejection function. At T-DXd 7th cycle, an infusion reaction occurred. A desensitization protocol was made and well tolerated. With time, hypersensitive symptoms were seen more often, needing heavier support medication and decreased infusion rates. Oral corticosteroids suspension was never possible, with an achieved minimum of prednisone 20 mg a day alternating with 10 mg each day. Extended steroid AE, like symptomatic G2 myopathy, requiring prednisone tapering and encouragement of physical activity, as well as bilateral senile cataract, resolved surgically, were seen. These events, although interfering with daily functioning, were easily managed.

After twenty-seven T-DXd cycles, neurological symptoms worsened (especially frontal headaches and dizziness), needing pain medication intensification and increased corticosteroid. Even so, response evaluation showed maintained CNS stable disease, with no distant metastasis. Lung micronodules were observed after SARS-COV2 infection, too small for a biopsy. After multidisciplinary discussion, it was assumed an infectious etiology, non-treatment related.

After thirty-three T-DXd treatments, disease progression was observed, with *de novo* lung and hepatic lesions. Biopsy confirmed distant disease metastasis, marking a progression-free survival of twenty-six months (2.2 years). At this point, CNS disease was still described as stable (according to RANO-BM criteria). The case was discussed in a multidisciplinary committee, and the patient was proposed to second-line treatment with tucatinib, trastuzumab and capecitabine.

At this point, the patient’s global status was still very good, with an ECOG-PS of 1. Neurological symptoms were medically controlled, enabling complete autonomy in daily life activities, with little impact on quality of life (QoL). Globally, T-DXd was well tolerated, with excellent and prolonged clinical and radiological response, having a huge impact on this patient’s survival and morbimortality.

## DISCUSSION

Oncological cases must be discussed within a multidisciplinary team, in order to achieve the best management and results, approaching the patient in a holistic and integrative way [9]. The rise of new drugs and therapeutic combinations in many BC settings [3] has made discussion between peers and shared decisions a fundamental step towards best BC care.

As HER2 positive expression is now an established biomarker for BC molecular typing, anti-HER2 antibodies have been consolidated as first-line treatment in both HER2-positive EBC and MBC [4, 10], especially considering the major positive results of antibody drugs, specially trastuzumab. In HER2-positive EBC, the addition of one year of adjuvant trastuzumab to CTx significantly improves long-term disease-free survival (DFS) and OS [11, 12]. Higher pathological complete response (pCR) rate with neoadjuvant dual HER2 blockade (trastuzumab/pertuzumab) plus CTx was also observed [13]. The combination of dual HER2 blockade (trastuzumab/pertuzumab) and CTx as first-line in HER2-positive MBC significantly prolonged progression-free survival (PFS) [14].

After this, new research was developed, aiming for maximal dose-effect and reduced toxicity, regarding other systemic treatments. In the last few years, target therapies have evolved, and ADC emerged as a stand-out option in BC. ADC combines antibodies (targeting tumor-specific antigens) and cytotoxic drugs with a linker, giving it the ability to reach tumor cells more precisely. This helps ADC maximize tumor cell injury while reducing normal tissue damage, especially when compared to standard CTx [15]. Trastuzumab emtansine (T-DM1, an ADC with humanized IgG1 monoclonal antibody (mAb) trastuzumab linked to DM1, a microtubule inhibitor) and trastuzumab deruxtecan (T-DXd, an ADC with an anti-HER2 IgG1 mAb linked to DXd, a topoisomerase I inhibitor) are now well-known ADC in BC, presenting robust data regarding disease outcomes and quality of life improvement [3, 15].

In EBC setting, the substitution of adjuvant trastuzumab for T-DM1 in HER2-positive patients with residual invasive disease after neoadjuvant CTx and anti-HER2 therapy showed benefits, decreasing the risk of recurrence or death [16].

Metastatic disease is a more complex setting, presenting with worse prognosis and poor quality of life. Systemic treatment toxicity can largely contribute to that, and a risk/benefit balance needs to be considered [3, 4].

HER2-positive MBC trials showed significantly prolonged PFS and OS with T-DM1 in patients previously treated with trastuzumab and a taxane, presenting less toxicity than the control arm [7].

After that, T-DXd has been shown to have significantly prolonged PFS when compared to T-DM1 in HER2-positive MBC previously treated with trastuzumab plus taxane. However, T-DXd toxicity profile was not negligible, with special attention to interstitial lung disease and pneumonitis [8]. Even so, both T-DM1 and T-DXd were well tolerated, had manageable adverse effects and no grade 4 or 5 events were observed [7, 8].

More recently, a new ‘HER2-low’ concept emerged (including IHC scores of 1+ and 2+ without amplification by an ISH test), having important implications in treatment sequencing. Almost half of previously categorized HER2-negative BC have an HER2-low expression, constituting an important biomarker to treatment selection. Even so, HER2-low BC does not seem to represent a different BC subtype, instead evolving and expanding triple-negative and HR+ HER2 negative BC therapy options [17]. In this HER2-low MBC setting, T-DXd has already shown significantly longer PFS and OS than CTx of physician’s choice [18], becoming a newer and much needed therapeutic option to these patients.

On the other hand, and regarding T-DXd positive signs even in HER2-negative (zero score), new studies are now ongoing within this subpopulation [19, 20]. More research is also needed in HER2-positive HR-positive BC, regarding possible combination of endocrine therapy plus T-DXd, its efficacy and toxicity [21].

BC is one of the most common causes of central nervous system (CNS) metastasis, including brain metastasis (BM) and leptomeningeal carcinomatosis (LMC). These are more frequent in triple-negative and HER2-positive BC, when compared to luminal subtypes [5, 6]. Therapeutic options for BM/LMC BC can be limited, especially concerning CNS and blood-brain barrier drug penetration [22]. In recent data, T-DXd showed effectiveness regarding overall response rate (ORR), PFS and OS in BC patients with BM and sustained systemic and CNS disease control in LMC [23–26]. However, there is still a lack of solid and robust evidence on T-DXd activity in CNS metastasis and real-world data could help mitigate this gap.

Approaching the case above, at the time of T-DXd proposal for this patient, the drug use was still strictly restricted by Portuguese regulatory institutions. Even so, evidence from clinical trials was already known [8], leading to previous Food and Drug Administration (FDA) and European Medicines Agency (EMA) T-DXd approval on MBC. Off-label cancer drugs use is generally discouraged, even so, in some cases, it can be supported by high level evidence based on robust clinical trials data. Since off-label treatments approval is usually established on a case-by-case basis, regulatory institution restrictions, reimbursement difficulties and impaired access between institutions may happen more often than desirable [27]. At the time of treatment initiation, limited data from previous subgroup analysis [23, 24] suggested potential intracranial activity of T-DXd, providing a rationale for its off-label use in this setting.

The case described is an excellent example of the paradigm shift we are witnessing with the introduction of ADCs in BC treatment. As efficacy and response rates are encouraging, the drug is well tolerated, adverse effects are manageable and quality of life improvement is achieved, these helps explain this type of drug´s worldwide success. Although some issues are still rising, especially in financial and accessibility fields, research towards novel targets and ADC indications are expected to lead to enhanced targeted cancer therapy in BC.
